# Effect of Drum-Drying Conditions on the Content of Bioactive Compounds of Broccoli Pulp

**DOI:** 10.3390/foods9091224

**Published:** 2020-09-02

**Authors:** Constanza Córdova, Juan P. Vivanco, Julián Quintero, Andrea Mahn

**Affiliations:** 1 Departamento de Ingeniería Química, Facultad de Ingeniería, Universidad de Santiago de Chile, Avenida Libertador Bernardo O’Higgins 3363, Estación Central, Santiago 9170019, Chile; constanza.cordova@usach.cl (C.C.); julian.quintero@usach.cl (J.Q.); 2 Centro de Investigación y Tendencias (CIyT), Área de Innovación y Desarrollo, Alfa Chilena Alimentos, Av. Las Américas 700, Cerrillos, Santiago 9230102, Chile; jpvivanco@gmail.com

**Keywords:** double-drum dryer, sulforaphane, phenolic compounds, ascorbic acid, antioxidant activity

## Abstract

This work studied the effect of drum-rotation frequency, drum temperature, and water-to-pulp ratio in a double-drum drier on the content of sulforaphane, glucoraphanin, total phenolic compounds, ascorbic acid, and antioxidant activity of broccoli pulp through a multilevel factorial design with one replicate. Drum-drying conditions did not significantly affect sulforaphane content, unlike glucoraphanin, however the poor adherence of broccoli pulp resulted in a final product with undefined shape and heterogeneous color. On the other hand, antioxidant activity was unevenly affected by drying conditions; however, drum-rotation frequency affected it in the same way that phenolic compounds and ascorbic acid, showing a concordant behavior. The ascorbic acid content decreased significantly after drying, and it was highly dependent on the experimental factors, resulting in a regression model that explained 90% of its variability. Drum-rotation frequency of 5 Hz, drum temperature of 125 °C, and water-to-pulp ratio of 0.25 resulted in an apparent increase of sulforaphane and phenolic compounds content of 13.7% and 47.6%, respectively. Drum drying has great potential to fabricate dehydrated broccoli-based foods with functional properties. Besides, since drum drying has low investment and operation costs, it represents a very attractive option for the industrialization of broccoli derivatives.

## 1. Introduction

Broccoli (*Brassica oleracea* var. *italica*) offers many health beneficial effects due to its high content of bioactive compounds, such as polyphenols, ascorbic acid, sulforaphane (and its precursor, glucoraphanin), and high antioxidant activity. In the last decade, sulforaphane gained great attention because of its outstanding health-promoting properties, such as acting as indirect antioxidant at cellular level and as a powerful cancer preventing agent, by inducing phase II detoxifying enzymes. The formation of sulforaphane occurs through the hydrolysis of glucoraphanin mediated by the enzyme myrosinase (EC 3.2.1.147), therefore processing conditions greatly influence the health benefits of broccoli. This encouraged research on exploiting these healthy properties by optimizing culture, harvest, and post-harvest processing conditions of broccoli [1]. The edible parts of the plant are the flowering immature heads, which suffer a rapid senescence process because they are harvested before physiological growth ends. Consequently, broccoli preservation is imperative. The most popular preservation method is storage at low temperature (chilled or frozen); however, this method entails high energy demand for storage and transportation. Dehydration appears as an attractive alternative. Different dehydration processes have been studied in order to preserve broccoli keeping its health promoting properties to the maximum. Reyes et al. [2] studied the dehydration of selenium-enriched broccoli particles in a pulsed fluidized bed dryer and found that drying impaired the antioxidant properties and selenium content of broccoli. They reported the operating conditions that minimize this impairment. Mahn et al. [3] investigated the effect of freeze-drying conditions on the content of bioactive compounds of broccoli florets. The authors found that freeze drying at atmospheric pressure resulted in the highest content of bioactive compounds. Mahn et al. [4] studied the effect of tunnel-drying conditions on sulforaphane content in broccoli florets and reported a maximum of 19% sulforaphane retention in the dry product. Karaaslan [5] described microwave-assisted drying of broccoli florets using phenomenological and empirical models but did not evaluate the effect of operation conditions on the content of bioactive compounds. Salim et al. [6] studied the continuous flow osmotic dehydration of broccoli stalk slices, focusing on the optimization of the hydrodynamic control of the system. Ferreira et al. [7] reported microwave-assisted drying of broccoli by-products as a pre-treatment before extraction of bioactive compounds. The authors reported that this method preserves polysaccharides and proteins and allows the recovery of phenolic compounds and glucosinolates at a significant level. No studies about the drum drying of broccoli are available so far. The use of this technology would probably result in a lower cost process than the technologies explored until now, such as tray, fluidized bed, and freeze drying [8].

Drum drying is used mainly for dehydrating fruit and cereals, and it has been poorly explored for vegetables so far. The double-drum arrangement is the most popular one in the food industry because it is suitable for a wide range of materials. Besides, this type of equipment offers better economics and a more efficient operation [9]. The main industrial applications of drum drying relate with dehydration of liquid or paste-like foods that do not require a specific shape as the final product, such as powders or flakes. Double-drum dryers have been applied to maize starches [10], potato flakes [11], watermelon pomace [12], apple peel [13], pomegranate peel [14] wheat and cornstarch gels [15], prune and tomato pomaces [16], and mango pulp [17]. Recently, Yamato et al. [18] studied the stability of mango flakes obtained in a rotary single-cylinder drum dryer. In drum drying, the material to be dehydrated forms a thin layer on the surface of the drum so that drying occurs by conduction [9]. The operation variables that affect the performance of a drum dryer are the medium heating temperature (usually superheated or saturated steam), which determines the temperature at the surface of the drum; speed of rotation; thickness of the layer, and the properties of the material to be dried, which in turn determine the degree of adherence of the material [18]. The effect of surface drum temperature and rotation frequency must be evaluated, since some bioactive compounds of broccoli, such as sulforaphane and ascorbic acid, are highly thermo labile. Besides, increasing water content to the pulp could probably affect the content of hydrophilic compounds, such as glucosinolates.

The aim of this work was to investigate the effect of the operating conditions in a double-drum dryer on the content of bioactive compounds and antioxidant activity of broccoli pulp. The variables under study were rotation frequency of the drums, drum surface temperature, and water content of the broccoli pulp. The bioactive properties considered in this study were the content of sulforaphane, glucoraphanin, total phenolic compounds, ascorbic acid, and antioxidant activity.

## 2. Materials and Methods

### 2.1. Raw Material

Broccoli heads (*Brassica oleracea* var. *italica*) cv. Imperial were donated by Agrocesar Ltd.a. (Curacaví, Región Metropolitana, Chile). Broccoli was washed immediately after reception, cut into 5 cm × 0.7 cm pieces, and 400 g were blanched at 60 °C for 12 min in a food processor (Vorwerk Gmbh & Co., Typ. Thermomix 31-1, Berkshire, United Kingdom) with a slow mixing function, aiming at maximizing sulforaphane content, as suggested in the literature [19]. After that, the blanched broccoli pieces were homogenized in the Thermomix (Vorwerk Gmbh & Co., Typ. Thermomix 31-1, Berkshire, United Kingdom) at 4500 RPM for 60 s at 40 °C, obtaining a homogeneous pulp. The pulp was mixed with water either in the ratio 0.25 kg water: 1.0 kg pulp or 0.50 kg water: 1.0 kg pulp, in order to improve the adhesion properties of the pulp. The broccoli pulps were homogenized in a Microcut concentric mill MC 15 (Stephan Machinery GmbH, Hameln, Germany) at 3600 RPM and 16 °C before drying. The complete procedure was repeated until we obtained 15 kg of each type of broccoli homogenate.

### 2.2. Drum Dryer

**Figure 1:**
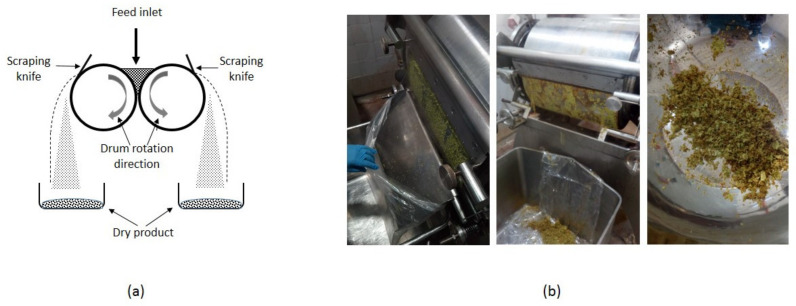
Double-drum dryer (**a**) operation scheme and (**b**) operation images.

The equipment consisted of a pilot double-drum dryer (Tummers R. Simon Dryers Technology Ltd., Nottingham, U.K.); each drum has 450 mm diameter and 450 mm length, located at the pilot plant of Alfa Chilena Alimentos Ltd.a. (Cerrillos, Santiago, Chile). The double-drum dryer was heated with saturated steam, so the surface drum temperature was adjusted by setting the input steam pressure. The temperature at the drums surface was verified with an infrared thermometer. The gap between the drums was set at 0.4 mm. Each of the 12 experimental runs considered a batch of 2.5 kg of broccoli pulp. Figure 1a shows a scheme of the drum dryer (frontal view), and Figure 1b shows images of the dryer operation.

### 2.3. Experimental Design and Statistical Analyses

Since adherence of the broccoli pulp was insufficient to conduct the drum drying, it was necessary to improve it by adding water at a certain water-to-pulp ratio. A complete multilevel factorial design with one replicate was applied to investigate the effect of the drum-rotation frequency, water-to-pulp (W:P) ratio, and drum surface temperature on some bioactive properties of broccoli. Table 1 shows the experimental matrix in standard order. The factors were chosen considering the controllable variables of the equipment, and the levels were chosen based on preliminary experiments (data not shown). The experiments were conducted randomly. Statistical analyses were made with Statgraphics™ Centurion XVII (Satgraphics Technologies, Inc., The Plains, Virginia, USA, 2013). The statistically significant effects were detected through ANOVA. Statistically significant differences between the final product and the initial pulps were assessed by Student’s *t* test. A 95% confidence interval was considered.

### 2.4. Analytical Determinations

#### 2.4.1. Chemicals

All chemicals were HPLC grade. Methylene chloride was purchased from J.T. Baker (Center Valley, PA, USA), anhydrous sodium sulfate, Folin-Ciocalteu reagent, 2,2-Diphenyl-1-picryhydrazyl, TPTZ, FeCl3·6H2O, and HPLC standards were purchased for Sigma-Aldrich (Schnelldorf, Germany). Organic solvents and acids were purchased from Merck (Darmstadt, Germany).

#### 2.4.2. Moisture Content

The moisture content determinations were carried out according to AOAC 920.151 [20] by dehydrating at 80 °C in a vacuum oven model 60,061 (Cole & Palmer, Holdpack, IL, USA) until constant mass. The average of three measurements was taken for calculations. All measurements were made in triplicate.

#### 2.4.3. Sulforaphane Content

Sulforaphane (SFN) was quantified by reverse phase HPLC, following the method reported elsewhere [21]. One gram of pulverized broccoli was extracted twice with 10 mL methylene chloride combined with 0.5 g anhydrous sodium sulfate. The equipment consisted in a HPLC with Diode Array Detector (DAD) (Shimadzu, Kyoto, Japan) provided with a C18 column (5 µm particle size, 250 × 4.6 mm) (Agilent Technologies, Santa Clara, CA, USA). The solvent was composed by 20% acetonitrile in water; changing linearly for 10 min to 60% acetonitrile and maintained at 100% acetonitrile for 5 min. The temperature was set at 30 °C, the flow rate was 1 mL·min^−1^, and the injection volume was 20 mL. Absorbance at 254 nm was recorded. Quantification was made by the method of external standard comparing the results with a sulforaphane standard curve. The measurements were made in triplicate. Results are expressed as mean ± standard deviation.

#### 2.4.4. Glucoraphanin Content

Glucoraphanin (GFN) was quantified by HPLC following the method published elsewhere [22]. 100 mg of pulverized broccoli were extracted twice with 1.5 mL of 70% methanol; incubated at 70 °C for 30 min with vortex agitation every 5 min; centrifuged, and the supernatant was collected. Methanol was removed in a rotary evaporator Stuart RE-300 (Cole-Parmer, Staffordshire, United Kingdom) to dryness at 30 °C under vacuum. The solid was resuspended in 1 mL ultrapure water and filtered through 0.22 µm syringe PVDF filters (Millex^®^-GV, Merck, Darmstadt, Germany). Quantification was conducted in a HPLC-DAD (Shimadzu, Kyoto, Japan) provided with a C18 column (5 µm particle size, 250 × 4.6 mm) (Agilent Technologies, Santa Clara, CA, USA). The mobile phase consisted of trifluoro acetic acid 0.1% (buffer A) and 0.1% trifluoro acetic acid in acetonitrile (buffer B). The elution gradient started with 0% B at 0–5 min, changing linearly to 5.1% at 8 min, reaching 99% B at 15 min, and 0% B at 20 min. The temperature was set at 30 °C, the flow rate was 1 mL·min^−1^, and the injection volume was 20 mL. Absorbance at 226 nm was recorded. Quantification was made by the method of external standard comparing the results with a sinigrin standard curve. Measurements were made in triplicate. Results are expressed as mean ± standard deviation.

#### 2.4.5. Total Phenolic Compounds

The content of total phenolic compounds (TPC) was determined in a spectrophotometer (Rayleigh mod UV1601 UV/VIS, Beijing, China) through the Folin-Ciocalteu method [23]. An amount of 180 µL extract and 90 µL Folin-Ciocalteu reagent (diluted 1:1) were added to 360 µL distilled water. The mixture was homogenized and left in the dark for 5 min. Then, 450 µL of a 200 g·L^−1^ sodium carbonate solution was added and left in darkness for 30 min. After that, samples were centrifuged at 12,000× *g* for 5 min to remove the precipitate, and absorbance at 750 nm was measured. The results were expressed as mg of gallic acid equivalents per gram of dry matter (mg GAE·g^−1^ DM). The measurements were made in triplicate and average values were reported.

#### 2.4.6. Ascorbic Acid Content

Ascorbic acid content was assessed by HPLC according to the method reported in literature [24]. Extraction was made directly in mobile phase; the mixture was centrifuged at 5000 rpm for 5 min and filtered through 0.45 µm PVDF syringe filters (Millex^®^-GV, Merck, Darmstadt, Germany). Sample injection volume was 20 µL. The equipment was a HPLC-DAD Shimadzu (Tokyo, Japan), and a reverse phase C18 column (5 µm, 250 × 4.6 mm particle size) (Agilent Technologies, Santa Clara, CA, USA) was used. The solvent system consisted in 20% methanol and 80% phosphate buffer (20 mM, pH 4.0). The flow rate was set at 1 mL·min^−1^, and detection wavelength was set at 240 nm. The column oven was set at 40 °C. All measurements were made in triplicate.

#### 2.4.7. Free Radical Scavenging Ability

The free radical scavenging ability (FRSA) was measured using the stable radical 2,2-Diphenyl-1-picryhydrazyl (DPPH) [25]. 40 µL vegetable extract (at 6 dilutions) were mixed with 1960 µL DPPH solution (6 × 10^−5^ M in methanol). The absorbance decrease at 515 nm was continuously recorded for 30 min. The DPPH·concentration in the reaction mixture at zero time and after 30 min was calculated by means of a calibration curve and the remaining DPPH· concentration was obtained. Results are expressed in Trolox equivalents. The measurements were made in triplicate, and average values were reported.

#### 2.4.8. Ferric Reducing Ability

The ferric ion reducing ability (FRAP) was measured following the protocol reported elsewhere [26]. FRAP reagent was obtained by mixing a solution of 20 mM FeCl_3_·6H_2_O with a solution of 10 mM TPTZ in 40 mM HCl and 0.3 M acetate buffer (pH 3.6) in the proportion 1:1:10 (*v:v:v*). A sample aliquot of 100 µL was mixed with 400 µL 80% methanol and 1 mL FRAP reagent, agitated and incubated at 37 °C for 30 min in the dark. Absorbance at 593 nm was recorded. All measurements were made in triplicate and average values were reported.

## 3. Results and Discussion

The effect of drum-rotation frequency, drum surface temperature, and water-to-pulp ratio was evaluated on the content of moisture, sulforaphane, glucoraphanin, total phenolic compounds, ascorbic acid, and antioxidant activity (FRSA and FRAP) in the dried product. The properties of the broccoli pulps are given in Table 2. Table 3 shows the results obtained from each experimental condition. The significant differences between the final product and the humid broccoli pulps are indicated with asterisks.

**Figure 2:**
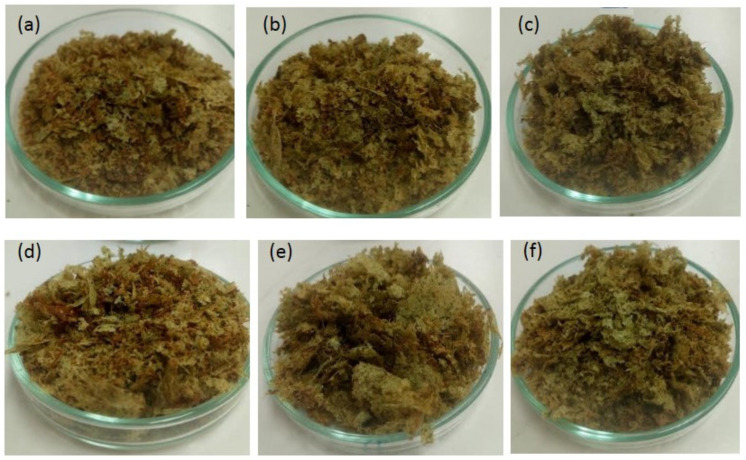
Images of the dehydrated product obtained in different conditions, according to Table 1. (**a**) Run 1, (**b**) run 2, (**c**) run 3, (**d**) run 4, (**e**) run 5, and (**f**) run 6.

Figure 2 shows images of the dried broccoli obtained in runs 1 to 6. The dried product has undefined shape and heterogeneous color. This is related with the low adherence of the pulp (as depicted in Figure 1b), which was composed only of broccoli florets and water. The adherence properties of the pulp can be improved by adding structuring compounds, such as maltodextrin, carboxy methyl cellulose, starch, or others [27,28]. The improvement of the adherence properties of the pulp falls outside the scope of the present work, however such study should be carried out in the future.

### 3.1. Final Moisture Content

**Figure 3:**
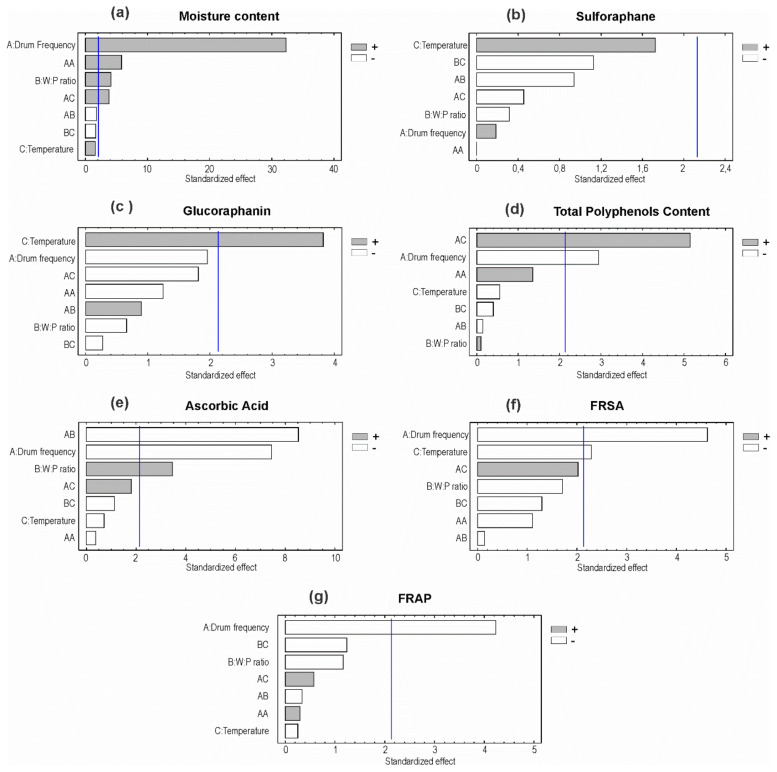
Standardized statistical effects of the experimental factors on the responses (Pareto charts); (**a**) Moisture content; (**b**) sulforaphane; (**c**) glucoraphanin; (**d**) phenolic compounds; (**e**) ascorbic acid, (**f**) FRSA = free radical scavenging ability and (**g**) FRAP = ferric ion reducing ability; A = drum-rotation frequency; B = water-to-pulp ratio; C = drum temperature.

The final moisture content varied between 5.9 ± 0.6% (run 7) and 26.3 ± 0.3% (run 9). The lowest moisture content obtained in this work is similar to that obtained in fluidized-bed drying [2] and lower than that reported for freeze drying of broccoli (9.7%) [29], suggesting that drum drying could give higher stability than freeze drying of broccoli. If the dried product is intended to be a dehydrated food ingredient, similar to a flour, the maximum moisture content should not exceed 15%, as stated by the Food and Drugs Administration (FDA) [30]. Then, the conditions used in runs 3, 6, 9, 11, and 12 would not be suitable for industrialization of this product. Lowest frequencies and the lowest W:P ratios resulted in lower final moisture contents. The statistical analysis indicates that the regression model explained 98.6% of the variability in moisture content. Figure 3a shows that drum-rotation frequency and water-to-pulp ratio significantly affected the final moisture content. Both factors exhibited a positive effect on this response, i.e., higher frequency, and higher W:P ratio produced higher final moisture content. This relates with the shorter drying time associated to higher frequencies. In addition, higher initial moisture content implies higher final moisture content at the same drying conditions. Contrary to what was expected, drum temperature had no significant effect on final moisture content. This result might owe to the narrow temperature range considered in the experimental design, which was established considering the industrial equipment limitations. Nevertheless, the interaction between temperature and drum frequency showed a significant positive effect on final moisture content.

### 3.2. Sulforaphane and Glucoraphanin Content

The blanching procedure used in this work corresponds to the optimized method to maximize SFN content in broccoli florets, as reported by [19]. In this way, the initial SFN content in the pulp before drying is significantly higher than that found in the fresh vegetable, and consequently, GFN content is lower than in unprocessed broccoli. In most runs (runs 2, 4, 6 to 10, and 12), sulforaphane content in the final product showed no statistically significant differences with the fresh pulp. Runs 1, 3, 5, and 11 showed a significant decrease of sulforaphane content (Table 3). The decrease of sulforaphane content may be attributed to thermal decomposition, considering the thermo lability of sulforaphane [31]. The regression model explained only 26.7% of the variability in sulforaphane content, then variations in sulforaphane content in the dried product cannot be attributed to the operation conditions considered in this study. Figure 3b shows that the experimental factors did not affect significantly sulforaphane content in the dried broccoli. Despite the fact that temperature had a positive effect on sulforaphane content, this effect was not significant. Considering sulforaphane thermo lability, this result may be related with the short time that the pulp was subjected to high temperatures and the narrow temperature range considered in this study. Then, drum-drying conditions did not affect significantly the sulforaphane content in the final product.

Table 4 shows a comparison of the maximum sulforaphane contents achieved in broccoli subjected to different drying methods using the same blanching conditions than in the present work. The highest retention was attained in freeze drying (129.7%) [29], however the high variability of the data suggests that those values are comparable with the maximum sulforaphane retention achieved in the present work (113.7%). Retention values above 100% may be related to an improvement in the extractability of the compounds from the food matrix due to changes in the food microstructure [32]. Besides, in this work no statistically significant difference was detected between the fresh pulp and the dry product obtained in run 2, therefore this apparent increase could also be interpreted as experimental deviation. Sulforaphane retention after convective and fluidized bed drying were by far lower than this value. Besides, freeze drying produced a final moisture content of 14.5%, which is above the recommended for vegetable flours. Drum drying resulted in a final moisture content of 13.0%, complying with the recommendations for this kind of products. Our results suggest drum drying as a promising dehydration process to preserve sulforaphane content in dehydrated broccoli, with lower operation and investment costs in comparison with freeze drying.

Glucoraphanin content in the dehydrated broccoli differed significantly from the content in the pulp in most runs. A significant increase in GFN content was observed in runs 2, 7, 8, and 10, while runs 1, 3, 4, and 6 resulted in a significant decrease. Drum temperature in runs 7, 8, and 10 was 128 °C, while in runs 1 to 6 it was 125 °C. Additionally, the statistical analyses show that temperature significantly affected glucoraphanin content in a positive way (Figure 3c). This may be attributed to an improvement in the extractability of intracellular compounds, an increase of solubility of GFN, and also to myrosinase denaturation at high temperatures, avoiding the conversion of glucoraphanin into sulforaphane. Drum-rotation frequency and W:P ratio did not significantly affect glucoraphanin content in the final product. The regression model derived from the statistical analysis explained 62.4% of the variability in GFN content. Although this value is relatively low for this kind of models, it confirms that operation conditions partly explain the behavior observed for this response.

The effect of drum-drying conditions on sulforaphane and glucoraphanin content showed different tendencies, contrary to what was expected. Since glucoraphanin is the substrate of myrosinase to yield sulforaphane, it was expected that an increase in sulforaphane implies a decrease in glucoraphanin content. However, our results contradict this hypothesis. This may be related with the combined effects of myrosinase partial denaturation, sulforaphane decomposition, and the extractability improvement due to microstructural changes in the food matrix during the drying process.

### 3.3. Total Phenolic Compounds

Drum drying produced an increased content of phenolic compounds, showing significant differences with respect to fresh pulp in 10 out of the 12 runs (Table 3). Besides, drum-rotation frequency had a significant negative effect on this response, i.e., a higher frequency decreases phenolic compounds content. A higher frequency implies shorter time of exposure to high temperatures (Figure 3d). The combined effect of temperature and drum frequency had a significant positive effect on TPC content. The increase in TPC content may be explained by the hydrolysis of phenolic compounds polymers, such as tannins, that probably increased because of exposition to high temperatures. Besides, tissue damage produced by grinding and heating of broccoli pulp facilitates the release of the phenolics polymers from the cell wall, thus making them available for their analytical quantification. Our results agree with the literature [34], where the highest polyphenols recovery from pomegranate was when the fruit was steamed, in comparison with high pressure and microfiltration processing. On the other hand, even though broccoli was subjected to tissue disruption before drying in order to obtain a pulp, at the inlet of the drum dryer the pulp passes through a narrow gap whose spacing determines the pulp layer thickness. Therefore, broccoli pulp is squeezed just before drying, producing higher tissue damage and thus facilitating the release of compounds from inside the vegetal cells. Additionally, most enzymes were probably inactivated due to thermal treatment (blanching and drying), and then polyphenols were not degraded by the action of polyphenol oxidase. Therefore, TPC content did not decrease after drying. The regression model explained 58.9% of the variability in TPC content, confirming that the variation of this response obeys to the drying conditions combined with physical and biochemical phenomena that were not explicitly considered in this study.

The maximum TPC retention obtained in this study was 147.6%, which corresponds to the highest value reported for broccoli drying, as shown in Table 4. Accordingly, drum drying seems the most adequate drying process to obtain dehydrated broccoli with a high content of total phenolic compounds.

### 3.4. Ascorbic Acid

The content of ascorbic acid was significantly lower than the content in the humid pulps in all runs. This may be attributed to thermal decomposition of ascorbic acid, agreeing with the literature [35]. The maximum retention of ascorbic acid was 70.6% (run 4), higher than that reported by Jin et al. [36] for broccoli dried at 50 °C (54% retention). Higher retention of ascorbic acid in drum drying, up to 90%, has been reported for mango pulp [18]. Drum-rotation frequency had a significant negative effect on ascorbic acid content, whereas water-to-pulp ratio had a significant positive effect on it (Figure 3e). Higher frequencies produced a decrease in ascorbic acid (AA) content, and temperature did not significantly affect AA content. This is in contradiction with expected since ascorbic acid is thermo labile, and low frequencies imply longer exposure time to high temperature. It can be inferred that the contact with the drum surface was too brief to produce thermal degradation of ascorbic acid. In addition, the temperature range considered in this study was relatively narrow, therefore no significant effect of this factor could be detected. On the other hand, water-to-pulp ratio had a significant positive effect on this response, probably because ascorbic acid is highly water soluble, and a higher water content in the pulp would improve its extractability. The regression model explained 90.6% of the variability in AA content, then this response was mostly determined by the operation conditions considered in this work.

### 3.5. Antioxidant Activity

FRSA showed no significant variation with respect to the initial pulps in runs 2 to 5 and 7 to 10. In run 1, there was a significant increase in FRSA, while in runs 6, 11, and 12, there was a significant decrease (Table 3). Drum-rotation frequency and temperature had significant negative effects on FRSA, then an increase of frequency or temperature produced a decrease in FRSA of the dried broccoli. Water-to-pulp ratio and the second order effects were not significant, as shown in Figure 3f. FRAP was significantly lower than the initial FRAP in runs 2, 3, 6, 8, and 12, while it was significantly higher in runs 4 and 11. In runs 1, 5, 7, 9, and 10, it showed no significant variations. Only drum-rotation frequency significantly affected FRAP in a negative way (Figure 3g). The other experimental factors had no significant effects on this response. It was expected that antioxidant activity (FRSA and FRAP) follows a similar tendency as TPC and ascorbic acid (AA) content, since both TPC and AA contribute to antioxidant activity. Drum-rotation frequency had significant negative effects on FRSA, FRAP, TPC, and AA content, showing that the four responses are related to each other. Temperature did not significantly affect FRAP unlike FRSA, probably because FRAP detects the metal chelating ability of antioxidant molecules, and polyphenols exhibit this capacity, but ascorbic acid does not. FRSA detects both free radical scavenging and metal chelating abilities, then the effect on ascorbic acid is reflected in FRSA, and not in FRAP.

## 4. Conclusions

Drum-drying conditions did not affect significantly the sulforaphane content in the final product. The maximum SF retention (113.7%) was attained at drum-rotation frequency of 5 Hz, drum temperature of 125 °C, and W:P ratio of 0.25 (run 2). This value is comparable to that obtained in freeze drying, and it is the highest value reported so far. The maximum TPC retention (147.6%) was obtained in the same conditions of W:P and temperature but drum frequency of 4.2 Hz (run 1), being the highest value reported for broccoli drying. Accordingly, drum drying seems the most adequate drying process to obtain dehydrated broccoli with high content of sulforaphane and total phenolic compounds. Ascorbic acid content was significantly lower in dried broccoli, and it showed high dependence of the drying conditions, with drum frequency affecting it negatively and water-to-pulp ratio affecting its content positively. Antioxidant activity (FRSA and FRAP) varied unevenly with the different drying conditions, probably because FRSA and FRAP reflect the contribution of several compounds that exhibit different antioxidant mechanisms. However, both FRSA and FRAP, as well as TPC and AA, were significantly affected by drum-rotation frequency in a negative way, thus showing the concordant behavior of these four responses. Overall, drum drying seems promising to exploit bioactive properties of broccoli and deliver them as part of a dehydrated food that could be used as an ingredient with functional properties. Besides, drum-drying technology is by far cheaper than other drying methods, such as freeze drying and tunnel drying. Then, this technology represents a very attractive option for the industrialization of broccoli derivatives.

## Figures and Tables

**Table 1:** Experimental design in standard order. Coded levels of experimental factors appear in parentheses. DF = drum frequency; W:P = water-to-pulp ratio; T = drum surface temperature.

Run	DF (Hz)	W:P Ratio	T (°C)
1	4.2 (−1)	0.25 (−1)	125 (−1)
2	5 (0)	0.25 (−1)	125 (−1)
3	6.3 (+1)	0.25 (−1)	125 (−1)
4	4.2 (−1)	0.50 (+1)	125 (−1)
5	5 (0)	0.50 (+1)	125 (−1)
6	6.3 (+1)	0.50 (+1)	125 (−1)
7	4.2 (−1)	0.25 (−1)	128 (+1)
8	5 (0)	0.25 (−1)	128 (+1)
9	6.3 (+1)	0.25 (−1)	128 (+1)
10	4.2 (−1)	0.50 (+1)	128 (+1)
11	5 (0)	0.50 (+1)	128 (+1)
12	6.3 (+1)	0.50 (+1)	128 (+1)

**Table 2:** Content of bioactive compounds of the pulps before drying.

Initial Content in the Pulps	W:P Ratio	
	0.25 (kg/kg)	0.50 (kg/kg)
Moisture content (g water/g wb)	93.3 ± 1.1	93.8 ± 1.1
Moisture content (g water/g dw)	14.0 ± 0.2	15.1 ± 0.2
Sulforaphane (mg/100 g dw)	151.2 ± 8.6	162.4 ± 8.3
Glucoraphanin (mg/100 g dw)	18.6 ± 1.3	20.8 ± 4.7
Total polyphenols (GAE/100 g dw)	397.6 ± 0.6	390.3 ± 17.7
Ascorbic acid (mg/100 g dw)	61.7 ± 7.1	65.9 ± 6.0
FRSA (TE/100 g dw)	300.1 ± 9.7	338.2 ± 19.6
FRAP (TE/100 g dw)	193.4 ± 3.4	173.7 ± 1.8

All measurements were made in triplicate; FRSA = free radical scavenging ability; FRAP = ferric ion reducing ability; dw = dry weight; wb = wet base; W:P = water-to-pulp ratio; GAE = gallic acid equivalents; TE = Trolox equivalents.

**Table 3:** Responses of each experimental run as described in Table 1.

Run	FMC (%)	X (g Water/g dw)	SFN (mg/100 g dw)	GFN (mg/100 g dw)	TPC (mg GAE/100 g dw)	AA (mg/100 g dw)	FRSA (TE/100 g dw)	FRAP (TE/100 g dw)
1	6.9 ± 0.5	0.07 ± 0.01	96.0 ± 0.5 *	14.6 ± 1.8 *	587.0 ± 8.5 **	19.0 ± 0.9 *	336.0 ± 2.1 **	197.0 ± 2.8
2	13.0 ± 2.2	0.15 ± 0.04	171.9 ± 2.4	24.9 ± 2.1 **	415.5 ± 29.0	24.3 ± 1.0 *	318.1 ± 8.2	155.0 ± 1.4 *
3	21.9 ± 0.3	0.28 ± 0.01	115.9 ± 4.2 *	14.1 ± 0.6 *	444.5 ± 3.5 **	14.7 ± 0.6 *	295.0 ± 5.3	150.0 ± 0.0 *
4	10.4 ± 0.1	0.12 ± 0.01	153.2 ± 7.1	15.3 ± 0.3 *	571.5 ± 6.4 **	43.6 ± 0.2 *	337.2 ± 1.4	181.5 ± 0.7 **
5	13.9 ± 1.8	0.16 ± 0.03	128.7 ± 15.2 *	21.2 ± 2.6	536.0 ± 1.4 **	26.1 ± 1.5 *	325.1 ± 22.8	175.0 ± 2.8
6	24.9 ± 1.5	0.33 ± 0.04	151.5 ± 34.7	15.1 ± 1.0 *	365.5 ± 36.1	10.8 ± 0.1 *	279.0 ± 17.1 *	147.0 ± 12.7 *
7	5.9 ± 0.6	0.06 ± 0.01	156.0 ± 5.5	32.0 ± 3.6 **	477.5 ± 37.5 **	14.3 ± 1.4 *	317.0 ± 23.1	194.5 ± 2.1
8	13.7 ± 1.1	0.16 ± 0.02	158 ± 17.3	24.1 ± 1.4 **	461.5 ± 20.5 **	22.9 ± 0.0 *	322.0 ± 10.7	152.5 ± 2.1 *
9	26.3 ± 0.3	0.36 ± 0.01	168 ± 10.2	19.8 ± 3.5	499.5 ± 34.6 **	22.9 ± 0.7 *	291.5 ± 17.9	175.5.5 ± 17.7
10	8.7 ± 0.5	0.10 ± 0.01	160.0 ± 7.6	27.6 ± 3.4 **	451.5 ± 7.1 **	42.1 ± 4.4 *	301.1 ± 14.8	168.5 ± 13.4
11	14.4 ± 0.2	0.17 ± 0.00	132.5 ± 5.0 *	20.2 ± 1.1	447.0 ± 7.1 **	18.4 ± 0.5 *	285.1 ± 9.3 *	178.5 ± 0.7 **
12	25.9 ± 0.2	0.35 ± 0.01	140.3 ± 20.0	23.1 ± 5.3	524.5 ± 0.7 **	10.9 ± 0.6 *	289.0 ± 16.1 *	125.5 ± 3.5 *

FMC = final moisture content in wet basis; X = final moisture content in dry basis; SFN = sulforaphane, GFN = glucoraphanin; TPC = total polyphenols; FRSA = free radical scavenging ability; FRAP = ferric ion reducing ability; TE = Trolox equivalents; AA = ascorbic acid; GAE = gallic acid equivalents; All analytical determinations were made in triplicate; * denotes significantly lower (*p* < 0.05); ** denotes significantly higher (*p* < 0.05) than the initial pulp.

**Table 4:** Comparison of sulforaphane and total phenolic compounds in broccoli subjected to different drying methods.

Drying Method	SFN Content (mg/100 g dw)				TPC Content (mg GAE/100 g dw)			
	Final Moisture Content (% wb)	Initial SFN	Final SFN	SFN Retention (%)	Final Moisture Content (% wb)	Initial TPC	Final TPC	TPC Retention (%)
Drum dryer ^†^	13.0	151.2 ± 8.6	171.9 ± 2.4	113.7	6.9 ± 0.5	397.6 ± 0.6	587.0 ± 8.5	147.6
Fluidized bed dryer	16.8	143.6 ± 5.4	26.6 ± 1.8	18.5 [33]	5.0 ± 0.0	329.6 ± 6.9	309.1 ± 14.5	93.8 [33]
Freeze dryer	14.5	41.8 ± 6.7	54.2 ± 16.3	129.7 [29]	6.5 ± 0.0	957.2 ± 5.7	674.6 ± 17.0	70.5 [29]
Tunnel dryer	8.1	104.0 ± 1.5	19.9 ± 2.4	19.1 [4]	6.2 ± 0.1	520.5 ± 21.2	117.5 ± 2.7	22.6 [4]

In all drying methods broccoli was subjected to the same pretreatment, as suggested in [19]; Initial = content before drying; final = content in the final dry product; SFN = sulforaphane; TPC = total phenolics compounds; GAE = gallic acid equivalents; dw = dry weight; wb = wet basis; ^†^ Data obtained in this study.
